# Emerging therapeutic strategies targeting extracellular histones for critical and inflammatory diseases: an updated narrative review

**DOI:** 10.3389/fimmu.2024.1438984

**Published:** 2024-08-14

**Authors:** Tinghang Yang, Jing Peng, Zhuyun Zhang, Yu Chen, Zhihui Liu, Luojia Jiang, Lunqiang Jin, Mei Han, Baihai Su, Yupei Li

**Affiliations:** ^1^ Department of Nephrology, Kidney Research Institute, West China Hospital of Sichuan University, Chengdu, China; ^2^ State Key Laboratory of Polymer Materials Engineering, College of Polymer Science and Engineering, Sichuan University, Chengdu, China; ^3^ Department of Rheumatology and Immunology, West China Hospital of Sichuan University, Chengdu, China; ^4^ Jiujiang City Key Laboratory of Cell Therapy, Department of Nephrology, Jiujiang No. 1 People’s Hospital, Jiujiang, China; ^5^ Med+ Biomaterial Institute of West China Hospital/West China School of Medicine, Sichuan University, Chengdu, China; ^6^ Med-X Center for Materials, Sichuan University, Chengdu, China

**Keywords:** extracellular histones, damage-associated molecular patterns, inflammation, histone-neutralization, heparin, blood purification

## Abstract

Extracellular histones are crucial damage-associated molecular patterns involved in the development and progression of multiple critical and inflammatory diseases, such as sepsis, pancreatitis, trauma, acute liver failure, acute respiratory distress syndrome, vasculitis and arthritis. During the past decade, the physiopathologic mechanisms of histone-mediated hyperinflammation, endothelial dysfunction, coagulation activation, neuroimmune injury and organ dysfunction in diseases have been systematically elucidated. Emerging preclinical evidence further shows that anti-histone strategies with either their neutralizers (heparin, heparinoids, nature plasma proteins, small anion molecules and nanomedicines, etc.) or extracorporeal blood purification techniques can significantly alleviate histone-induced deleterious effects, and thus improve the outcomes of histone-related critical and inflammatory animal models. However, a systemic evaluation of the efficacy and safety of these histone-targeting therapeutic strategies is currently lacking. In this review, we first update our latest understanding of the underlying molecular mechanisms of histone-induced hyperinflammation, endothelial dysfunction, coagulopathy, and organ dysfunction. Then, we summarize the latest advances in histone-targeting therapy strategies with heparin, anti-histone antibodies, histone-binding proteins or molecules, and histone-affinity hemoadsorption in pre-clinical studies. Finally, challenges and future perspectives for improving the clinical translation of histone-targeting therapeutic strategies are also discussed to promote better management of patients with histone-related diseases.

## 1 Introduction

Histones, the main protein component of nucleosomes, can be divided into core histones (H2, H3, H4) and linker histones (H1) according to their function. Histones play a crucial role in regulating gene expression under physiological conditions (1). Due to their high proportion of amino acids with alkaline side chains, histones normally exhibit cationic properties that contribute to maintaining the well-organized structure of chromatin.

However, histones can cause harmful host response under certain pathological conditions. Once a pathogenic attack or sterile inflammation occurs, neutrophils are activated to form neutrophil extracellular traps (NETs), followed by the citrullination of histones by peptidylarginine deiminase 4 (PAD4), which further leads to the decondensation of chromatin and the release of extracellular histones (also known as circulating histones) and other histone-containing complexes (nucleosomes, NETs, etc.) into the blood circulation (2). In addition, numerous inflammatory mediators can activate innate immune cells including macrophages and induce massive tissue injury that also incur the release of extracellular histones. During the past decade, extracellular histones have been identified as a new group of damage-associated molecular patterns (DAMPs) that significantly induce hyperinflammation (3, 4), thrombocytopenia (5, 6), platelet aggregation (7), coagulopathy (8), endothelial cell death (9), and organ dysfunction (10, 11). Clinical studies further show that extracellular histones are associated with disease severity and mortality in patients with critically ill or inflammatory conditions such as sepsis (12, 13), septic shock (14), acute pancreatitis (13), ischemia–reperfusion injury (15, 16), acute liver failure (17), vasculitis (18), autoimmune arthritis (19), SARS-CoV-2 infection (20), and acute respiratory distress syndrome (ARDS) (21–24). Additionally, evidence has indicated that extracellular histones are capable of binding and inducing the aggregation of low-density lipoprotein and are strongly associated with the progression of atherosclerosis (25, 26). Extracellular histones also play a vital role in cancer dissemination, monitoring, and tumorigenesis in both hematologic malignancies and solid tumors (27). Furthermore, extracellular histones can also aggravate autoimmune arthritis by inducing lytic cell death in synoviocytes and macrophages through electrostatic interactions (19). For example, in patients with trauma-associated lung injury, nondegraded circulating histone elevated immediately from 10 to 230 μg/mL within 4 h and peaked at about 24 h (10). Other evidence showed the concentration of histones in serum and interstitial fluid was significantly increased in several disorders, such as sepsis, ARDS, systemic inflammatory response syndrome, cerebral stroke, trauma, liver dysfunctions after donor hepatectomy, and tumors (10, 25, 28–32), and was positively associated with the severity of organ failure and mortality (10, 28, 30, 33, 34).

Citrullination, the most discussed modification of histones, is the posttranslational conversion caused by PAD4 that leads to the decondensation of chromatin during NETosis. It is reported that that histone citrullination facilitates the translocation of NF-κB to the nucleus, which increases the release of proinflammatory cytokines tumor necrosis factor (TNF)-α and IL (interleukin)-1β (35). Wang et al. (36) reported that the serum levels of citrullinated histone 3 (citH3) were increased in a dextran sulfate sodium-induced ulcerative colitis murine model, while Cl-amidine (a PAD4 inhibitor) or PAD4 genetic knockout successfully alleviated the clinical colitis index, intestinal inflammation, and barrier dysfunction. Recently, many studies have collectively shown that CitH3 is significantly elevated in septic (37–39), ischemia-reperfusion (40), and abdominal aortic aneurysms (41) murine models and patients. CitH3 is more specific, persistent and sensitive than procalcitonin and inflammatory cytokines (37, 38, 42). Hence, increasing attention has recently been given to the multiple roles of citrullinated histones in disease diagnosis and prognosis (13, 38, 41, 43, 44). Tian Y et al. found that CitH3 was significantly increased in septic patients compared to healthy volunteers (101.5 pg/mL vs 8 pg/mL, p<0.0001) (38), and CitH3 level above 39 pg/mL correlated with higher disease severity and poorer prognosis patients with septic shock (38). Interestingly, Wang et al. (44) recently elucidated that the serum levels of citH3 in dermatomyositis patients were lower than those in healthy individuals (6.6 ng/mL vs. 33.6 ng/mL). This is due to the facts that not all the NETs-derived histones are citrullinated, and that citrullination is not a common characteristic of NETs (45, 46). Nakazawa et al. and Furubeppu et al. further demonstrated that therapy targeting NETs alone could not completely inhibit the remote organ dysfunction caused by free histones in systemic inflammation in ischemia/reperfusion injury (11, 40). Accordingly, emerging therapeutic strategies targeting extracellular histones for critical and inflammatory illnesses have arisen during the past decade.

Although the roles of extracellular histones in mediating systematic inflammation, thrombotic microangiopathy and disseminated intravascular coagulation, complement activation, and vascular endothelial dysfunction have been summarized in detail (47–51), reviews on histone-targeted therapy are lacking. In this review, we first update our latest understanding of the underlying mechanisms of histone-induced deleterious effects, and then summarize the latest advances in histone-targeting therapy strategies with heparin, anti-histone antibodies, histone-binding proteins or molecules, and histone-affinity hemoadsorption to shed light on novel therapeutic strategies for histone-related critical and inflammatory diseases.

## 2 Understanding the multiple roles of histones in critical and inflammatory diseases

**Figure 1:**
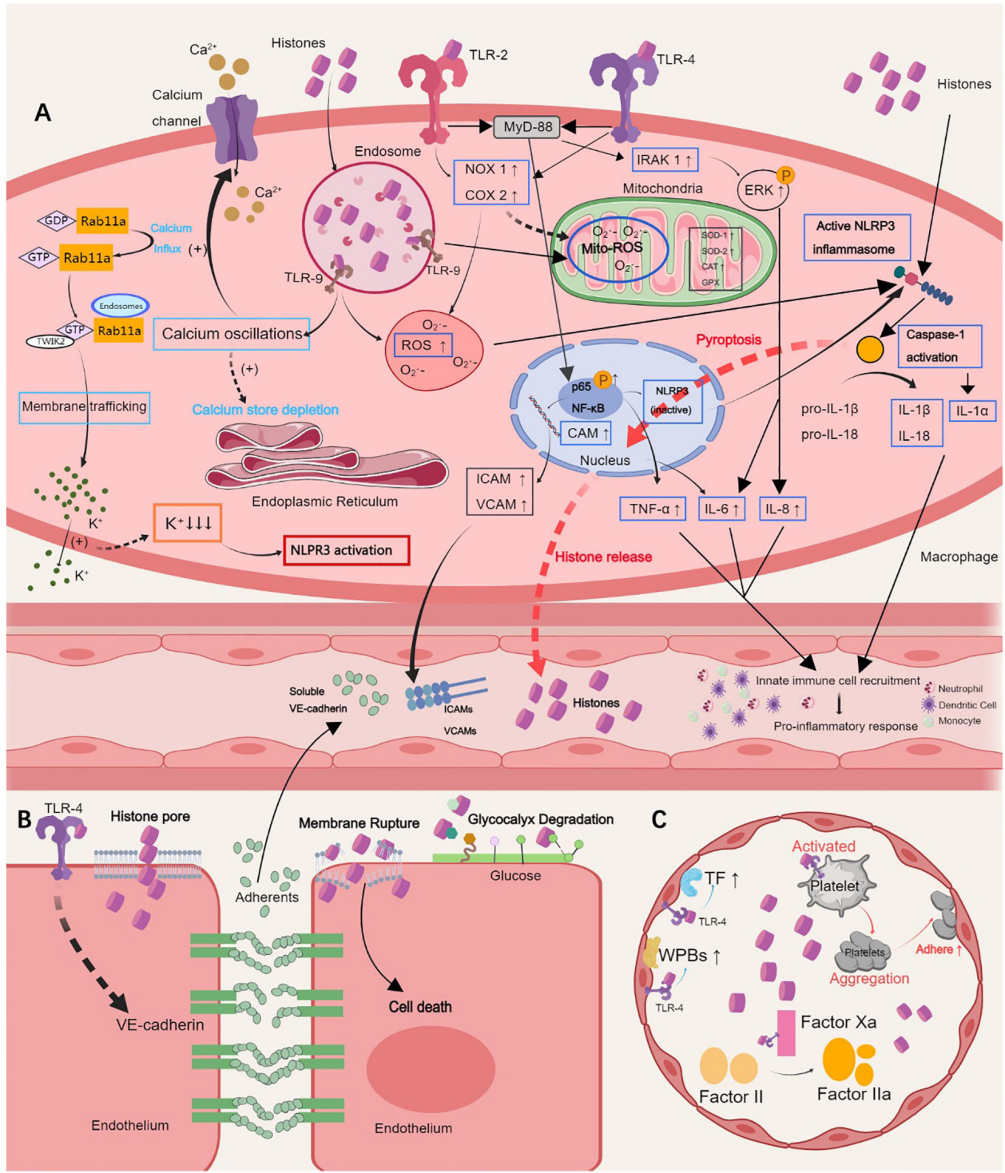
Pathological roles of extracellular histones. **(A)** Histones induce hyperinflammation by activating the TLR 2/4/9 or NLRP3 inflammasome pathways, which results in the release of proinflammatory cytokines, such as IL-6, IL-1α, IL-8, IL-1β, IL-18, and TNF-α, calcium oscillation, and cell pyroptosis, and further augments innate immune cell recruitment in the blood. **(B)** Histones sense TLR-4 to induce endothelial dysfunction, which is characterized by endothelial glycocalyx degradation, intercellular adherents destruction and vascular leakage, ultimately leading to inflammatory exudation, tissue edema and organ failure. **(C)** Histones self-recognizing with TLR-2 and TLR-4 promote histone-mediated platelet activation and aggregation and thrombocytopenia in a platelet-dependent manner. The expression of tissue factor and Weibel-Palade bodies is further induced by activating endothelial cells, causing thrombosis. TLR, Toll-like receptor; NLRP3, NOD-like receptor family pyrin domain-containing 3; IL, interleukin; TNF, tumor necrosis factor; ICAM, intercellular adhesion molecule; VCAM, vascular cell adhesion molecule. This picture was generated using MedPeer software.

The fine structure and physiological function of histones have been well-studied in recent decades. However, once histones are exposed to the extracellular environment, they exert significant cytotoxic and proinflammatory activities in a dose- and time-dependent manner (52). Recently, Riehl et al. (53) reported that histones are released in a temperature-dependent manner during reperfusion in renal transplantation. Interestingly, not all the subunits exhibited the same cytotoxicity. Numerous studies have indicated that subunits H3 and H4 are the main contributors to proinflammation, procoagulation (54), fibrinolysis (55) and endothelial injury (46). The detailed pathological roles of extracellular histones in critical and inflammatory diseases are discussed below and summarized in **Figure 1**.

### 2.1 Histone-induced hyperinflammation

Histones are released during necrotizing tissue damage and NETosis and lead to a domino reaction in inflammation in sepsis, acute liver failure, ARDS, etc. (56) Recent data have indicated that streptococcal inhibitor of complement, a protein released by *Streptococcus pyogenes*, can bind to histones to form large aggregates that abolish the antimicrobial function and prohemolytic function induced by histones. However, the proinflammatory effect was boosted (57). *In vitro*, direct administration of histones in the whole blood was significantly associated with thrombocytopenia and hyperinflammation in a dose-dependent manner (58).

#### 2.1.1 Activation of Toll-like receptor pathways

Many studies have verified that histones interact with Toll-like receptors (TLRs), especially TLR4 and TLR2, to cause the production of proinflammatory cytokines (IL-6 and TNF-a) via MyD88-dependent pathways (11, 57, 59–61), to trigger calcium oscillations (62), and to activate platelets that drive the augmented immune response resulting in tissue injuries (60, 63, 64). Recent data also indicated that the NOD2-VSIG4/NLRP3 (65), and TLR4-NF−kB/CAM (66) pathways may also be involved in histone-mediated inflammation by increasing the expression of IL-1β, IL-18, TNF-α, and IL-6 (67–69). Moreover, Zhang et al. (70) illustrated that the interaction between mitochondrial formyl peptides or mitochondrial DNA and TLR9 activated human polymorphonuclear neutrophils, which may ultimately contribute to histone-mediated systemic inflammatory response syndrome. Interestingly, Abrams et al.indicated significant MPO release in histone-associated inflammation, although histones did not directly simulate ROS production.

#### 2.1.2 Activation of NOD-like receptor family pyrin domain-containing 3 inflammasome

The NOD-like receptor family pyrin domain-containing 3 (NLRP3) inflammasome is activated and assembled in response to diverse damage stimuli, including DAMPs, lysosomal destruction, mitochondrial dysfunction, reactive oxygen species, and toxin-induced pore formation in cell membranes (71). As major DAMPs, extracellular histones significantly contributed to NLRP3 inflammasome activation and subsequent pyroptosis during sepsis (65) and acute lung injury (72). Jiang et al. (30) demonstrated that extracellular histones promoted alveolar macrophage pyroptosis through the NLRP3 inflammasome pathway, which aggravated inflammation in ARDS. The administration of MCC950, a NLRP3 inhibitor, particularly suppressed histone-induced macrophage pyroptosis and reduced the serum levels of IL-1β and IL-18, thus alleviated ARDS-related lung inflammation in murine models (29). The authors further showed that histone-induced NLRP3 activation in alveolar macrophages during sepsis was associated with increased TWIK2-dependent potassium efflux (73). Furthermore, Li et al. found that extracellular histones augmented heat stroke-induced hepatocyte pyroptosis and liver injury both *in vitro* and *in vivo* in a dose- and time-dependent manner via the TLR9-NLRP3 pathway (74). Likewise, histones were found to mediate the activation of the NLRP3 inflammasome and pyroptosis in endothelial cells to cause endothelial dysfunction (75).

#### 2.1.3 Other inflammatory pathways

Extracellular histones can also induce adipose tissue inflammation, which further contributes to metabolic dysfunction. Recently, Roos et al. (76) found that, in a murine polytrauma model, serum levels of histones significantly increased accompanied by an inflammatory response in white adipose tissue. Specifically, the histone-evoked inflammatory response in human adipocytes was mediated via the MYD88-IRAK1-ERK signaling axis (76). Moreover, histones can induce lytic cell death in human adipocytes executed independently of caspases and RIPK1 activity (76). These results suggest that preventing adipose tissue inflammation and adipocyte death by targeting extracellular histones in patients with polytrauma may help minimize posttraumatic metabolic dysfunction.

Histones are also implicated in the molecular pathogenesis of interstitial lung disease. Riehl et al. (53) recently found that the level of extracellular CitH3 was significantly increased in cell-free bronchoalveolar lavage fluid of patients with idiopathic pulmonary fibrosis than in that of healthy controls. Histones first activated platelets to release TGFβ1, which signaled through the TGFbRI/TGFbRII receptor complex on LysM+ cells to antagonize macrophage-derived IL-27 production (53). Neutralizing histones with monoclonal anti-histone H2A/H4 antibodies reduced the severity of experimental pulmonary fibrosis in mice.

Extracellular histones can mediate periodontitis by potentiating IL-17-mediated inflammation. Kim et al. recently showed that the levels of extracellular histones in the blood and local lesions of severe periodontitis patients were significantly increased (77). In an established periodontitis animal model, the authors demonstrated that histones triggered the upregulation of IL-17/Th17 responses, and bone destruction (77).

### 2.2 Histone-induced endothelium dysfunction

Among the different tissues affected during critical illnesses such as sepsis and pancreatitis, the endothelium is one of the most affected, as it is the first line of exposure to stimuli. The endothelium actively participates in and is affected by inflammatory progression. In particular, endothelial cells initiate coagulation by releasing factors that control platelet adhesion and blood clotting, magnifying the immune response by linking local and systemic immunoreactions, and regulating vascular tone and blood pressure (78). Data has proposed that endothelium dysfunction may be the primary cause of multiple organ dysfunction syndrome (MODS) and a crucial contributor to mortality in critically ill patients (79). In this regard, counteracting endothelial injury shows good prognostic characteristics.

Comprehensive studies of the effects of histone subunits on endothelium injury emphasize the pathological importance of subunits H3 and H4 (46, 80, 81), while the H1 and H2 subunits do not contribute to endothelial cytotoxicity (82). Recently, Osca-Verdegal et al. (39) demonstrated that citrullinated histones had less cytotoxic effects on endothelial cells than did free histones. Nevertheless, citrullinated histones still affect the inflammatory response and regulatory endothelial mechanisms (39). Histones significantly contribute to endothelial cell death, autophagy or apoptosis in a dose-dependent manner and mediate the destruction of cell-cell adherens junctions.

#### 2.2.1 Histone-induced endothelial cell death, autophagy and apoptosis

The dose threshold for histone-mediated endothelial dysfunction remains controversial due to endothelial variation (83). Generally, a high dose of histones can ubiquitously cause severe endothelial damage, resulting in a remarkable reduction in cell viability via direct binding between histones and phospholipid–phosphodiester bonds on the cell membrane, which leads to high permeability and the activation of calcium ion influx (48, 84). Although histones exhibited high affinity with glycosaminoglycans, especially heparan sulfate, the removal of heparan sulfate showed little protective effects against histone cytotoxicity, suggesting that the combination between glycosaminoglycans with histones might not be necessary for histone-mediated cytotoxicity (85). Importantly, Meara et al. (85) indicated that the addition of histones markedly reduced the liftime of lipid bilayers, verifying the direct cytotoxicity of histones. In contrast, low-dose histones induce autophagy and apoptosis in endothelial cells via mammalian target of rapamycin signaling (86).

#### 2.2.2 Histone-induced dysregulation of endothelial cell-cell adhesion

Although a high dose of histones (>100 µg/mL) can directly cause endothelial cell death (86), plasma levels of extracellular histones in patients are far below this threshold in most pathological conditions. Increasing evidence indicated that histone could cause endothelial barrier dysfunction beyond endothelial cytotoxicity. For instance, plasma concentrations of endothelial integrity-associated molecules, including syndecan-1, sphingosine-1-phosphate and soluble VE-cadherin, were significantly altered in severe septic patients (87, 88), suggesting that alterations in intracellular adhesion molecules might also play a crucial role in barrier dis-integrity and disease progression. More specifically, extracellular histones could destroy cell-cell adherents junctions, such as VE-cadherin (80, 89, 90), occluding (91) and zonular occludens 1 (84), and reorganize the cytoskeleton with increased F-action stress fibers to disrupt the endothelial barrier and directly exacerbate inflammatory damage (88).

Ramasubramanian et al. (82) compared the effects of various histone subunits (H1, H2A, H2B, H3, and H4) on human pulmonary endothelial cell permeability and the inflammatory response. Their results showed that histone H3 and H4, but not H1, H2A, or H2B, caused an increase in endothelial cell permeability accompanied by the disassembly of adherens junctions in a dose-dependent manner via a TLR4-dependent mechanism. Moreover, at higher doses, histones H3 and H4 activated the NF-kB inflammatory cascade and upregulated the expression of the endothelial adhesion molecules intercellular cell adhesion molecule 1 (ICAM1), vascular cell adhesion molecule 1 (VCAM1), and E-selectin and inflammatory cytokines. Similar findings were observed in other studies by Kim et al. and Pérez-Cremades et al. (66, 80). Notably, the elevated expression of adhesion molecules on the endothelial cell surface may increase the rolling, adherence, and transmigration of leukocytes into the underlying tissue.

Extracellular histone-induced endothelial damage further triggers severe pulmonary hemorrhage (10, 92). Transglutaminase 2 is abundant in endothelial cells, plays an essential role in promoting endothelial sprouting and the migration of vascular mesenchymal cells into endothelial cells (92). Mizuno et al. recently reported that, in a histone-induced acute lung injury animal model, transglutaminase 2 prevented C57BL/6J mice from histone-induced pulmonary hemorrhage by promoting fibrin deposition and adhesion of platelets to endothelial cells to restore endothelial barrier (92).

### 2.3 Histone-induced coagulopathy

Histones, as major DAMPs, promote thrombosis in a platelet-dependent manner. Histones bind to platelets, inducing platelet aggregation and thrombocytopenia (6) through self-recognition of TLRs, especially TLR2/TLR4 (93, 94). Verdegal et al. found a clear correlation between histones and total prothrombin, with the hypothesis that the histone-induced activation of the endothelium deprived the ability of platelets to adhere to the endothelium, which led to a longer time for clot development (39, 46). Histones can also promote endothelial cell activation, induce the expression of tissue factors (54) and Weibel-Palade bodies (1.46-fold) in a caspase dependent, calcium dependent, and charge-dependent manner, and thus cause thrombocytopenia (48, 95, 96). Additionally, histones can induce platelet polyphosphate release and phosphatidylserine exposure, which leads to the intrinsic coagulation activation in an FXII-dependent and TLR2/4-dependent manner (94, 97, 98).

Moreover, Michels et al. noted that the levels of histones and Weibel-Palade bodies were positively correlated with inflammation (95), suggesting that the histone-Weibel-Palade bodies axis might link coagulopathy to inflammation. Moreover, histones could also activate impairing thrombomodulin-dependent protein C (99) and were crosslinked to fibrin by FXIIIa (100), leading to increased thrombin generation and promoted fibrinolytic resistance.

In summary, histones markedly disrupt the fine balance and crosstalk between coagulant, anticoagulant, and inflammatory pathways to augment the procoagulant phenotype. Although the exact mechanism by which histones trigger thrombosis remains open to debate, it is well recognized that inflammation, thrombosis, and endothelial injury form an interactive network that contributes to multiorgan dysfunction or even death.

### 2.4 Histone-induced neuroimmune responses

During the past decade, it has been well established that histones exert neurotoxic effects that contribute to neuroimmune responses (101). For instance, Da Silva et al. demonstrated that histones could both damage neurons in a TLR4-dependent manner and alter the neuroimmune functions of glial cells, as evidenced by the reduced phagocytic activity of BV-2 microglia after LPS stimulation (102). Similarly, McRae et al. showed that both linker histone H1 and core histone H3 induced proinflammatory activation of microglia-like cells by upregulating the secretion of NO and cytokines, including interferon-γ-inducible protein 10 and TNF-α, through the TLR pathway (103). Accordingly, targeting extracellular histones to inhibit their neurotoxic activities may represent a potential strategy for combating neurodegenerative diseases that are characterized by the adverse activation of microglia and neuronal death.

### 2.5 Histone-induced vascular calcification

Vascular calcification is a major risk factor for cardiovascular events and is associated with a poor prognosis in patients with chronic kidney disease (104, 105). Vascular calcification is often characterized by the transformation of vascular smooth muscle cells into cells with osteoblast-like characteristics. Hoshino et al. recently reported that extracellular histones intensified calcium phosphate-dependent calcification by decreasing the expression of vascular smooth muscle cell marker genes while simultaneously increasing the expression of osteoblast marker genes (106). Histones could also induce inflammation and senescence in vascular smooth muscle cells by activating the AMPK/FOXO4 signaling pathway (107).

### 2.6 Histone-induced cardiac dysfunction

Histones significantly contribute to sepsis-associated cardiac dysfunction. Alhamdi Y et al. first demonstrated that histones induced profound calcium influx and overload in cultured cardiomyocytes, with dose-dependent detrimental effects on intracellular calcium transient amplitude, contractility, and rhythm, suggesting that histones directly affect cardiomyocyte function adversely (52). In histone-infused C57BL/6 mice model, a moderate sublethal histone dose of 30 mg/kg caused left ventricular contractile dysfunction characterized by a reduced ejection fraction and prolonged relaxation time. However, at high doses (≥ 60 mg/kg), histone administration led to pulmonary vascular obstruction, which further induced an increase in right ventricular pressure and dilatation. More recently, the same research group reported that histone-induced cardiac contractility depression was associated with protein kinase C alpha activation and troponin phosphorylation (108). Accordingly, blocking protein kinase C alpha significantly abrogated histone-induced deterioration in peak shortening, duration and the velocity of shortening and re-lengthening of cardiomyocyte contractility. These findings suggest that targeting circulating histones has potential translational benefits for critically ill patients with cardiac dysfunction and elevated plasma histone levels.

### 2.7 Histone-induced acute lung injury

The lung microvasculature is predominantly affected once histones are released into the blood circulation. Histones are toxic to cultured pulmonary endothelial cells, and histone levels are associated with acute lung injury and mortality (3). Extracellular histones could incur endothelial injury via the multiple pathways mentioned above, causing microvascular thrombosis, lung edema, and pulmonary hemorrhage. Recently, Fu et al. reported that histones could directly induce pulmonary syndecan-1 degradation via the heparinase pathway to cause a hyperpermeability of the pulmonary endothelium, as evidenced by collapsed alveoli, thickened alveolar walls, and obvious infiltration of neutrophils, lymphocytes, and erythrocytes in the alveoli in histone-infused murine models of ARDS (109). Moreover, histones can induce hyperinflammation and lung injury through activating either caspase-1 dependent inflammasome pathway, TWIK2-dependent potassium efflux or TLR4-dependent mechanisms (68, 73, 80). Accordingly, the administration of TLR-4 inhibitors significantly alleviated histone-induced vascular leakage and reduced the protein content and total cell and polymorphonuclear cell counts in bronchoalveolar lavage fluid in acute lung injury murine models (80, 82).

### 2.8 Histone-induced liver and kidney injury

During ischemic-reperfusion injury, dying hepatocytes and tubular epithelial cells release histones locally, promoting microvascular and parenchymal injury (3). Interestingly, the intraperitoneal administration of probiotics (200 mg/kg) significantly decreased the activation of the NF-kB pathway, decreased the mRNA levels of TLR-4, and improved the cell apoptosis in kidney tissues in an ischemia-reperfusion-induced acute kidney injury model (110, 111). Moreover, Li et al. reported extracellular histones could exacerbate heat stroke-induced liver injury by triggering hepatocyte pyroptosis and liver injury via the TLR9-NLRP3 pathway (74), stimulate collagen expression *in vitro* and promote liver fibrogenesis in a mouse model via the TLR4-MyD88 signaling pathway (112). Histone neutralization with an anti-histone antibody reduced postischemic liver and kidney injury (3).

## 3 Emerging anti-histone therapeutic strategies for critical and inflammatory diseases

**Figure 2:**
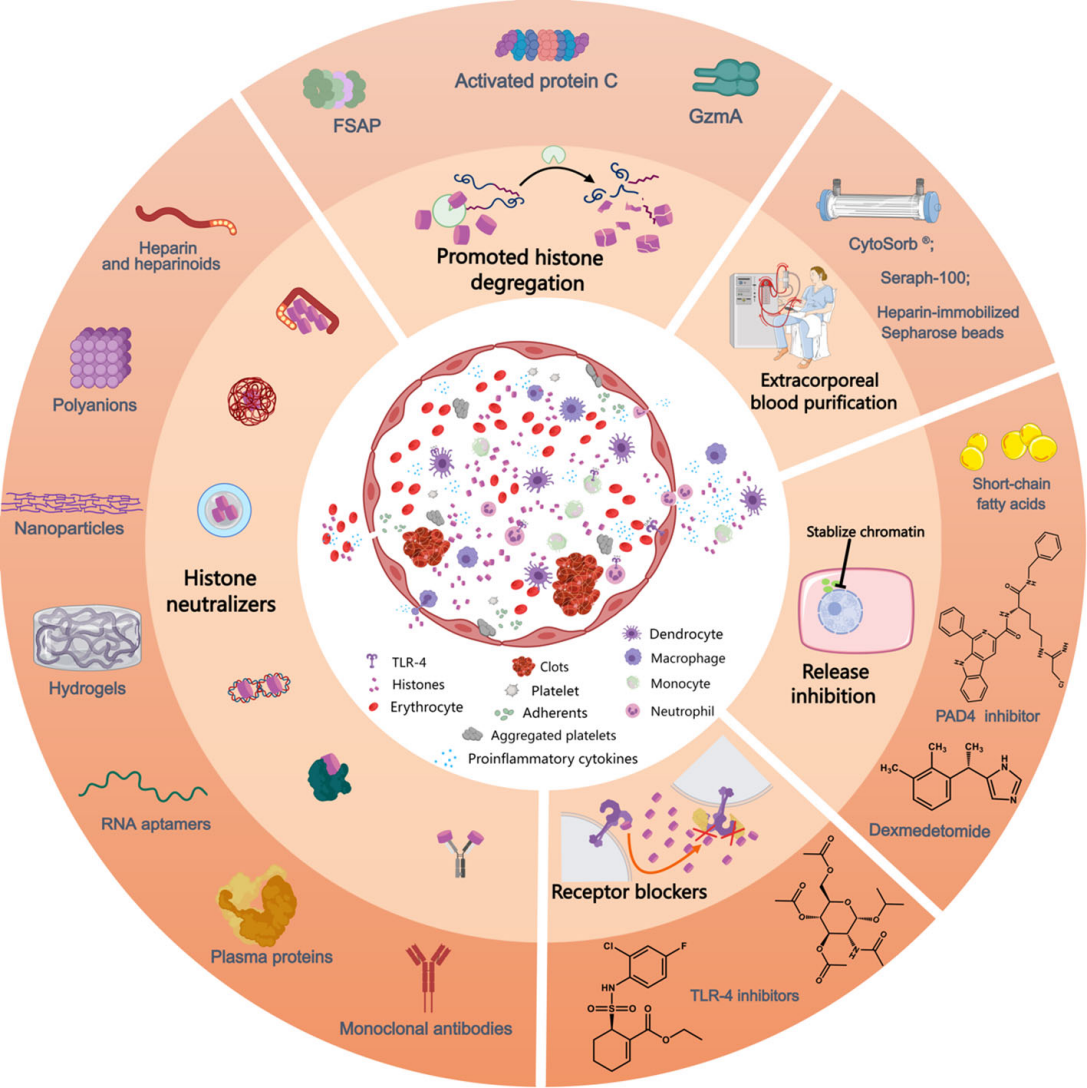
Mechanism of action of currently available anti-histone therapeutic strategies in preclinical studies. Extracellular histones exposed to the circulation induce the activation and aggregation of platelets, formation of clots, release of proinflammatory cytokines and recruitment and adhesion of innate immune cells, processes that can be inhibited by the use of histone-neutralizers, such as heparin and its heparinoids, polyanions, nanoparticles, hydrogels, monoclonal antibodies, RNA aptamers, plasma proteins (albumin, pentraxin-3, C-reactive protein, osteopontin, etc.), etc. Furthermore, various histone-interference strategies, including the promotion of histone degregation (FSAP, activated protein C, and GzmA) and extracorporeal blood purification for histone removal, decrease extracellular histone levels. Lesional histones induce barrier disintegrety, cell death and inflammatory reactions, processes that can be inhibited by related receptor blockers. Additionally, the inhibition of NET release by PAD4 inhibitors prevents the release of histones from neutrophils and reduces associated inflammation. Likewise, the administration of short-chain fatty acids and dexmedetomide lower histone levels in circulation. FSAP, factor VII-activating protease; GzmA, granzyme A; PAD4, peptidylarginine deiminase 4; TLR, toll-like receptor. This picture was generated using MedPeer software.

As outlined above, extracellular histones are major mediators of tissue injury and organ dysfunction. Accordingly, histones have become therapeutic target candidates for many critical or hyperinflammatory diseases in which inflammation and microcirculation disturbance play crucial pathological roles. In this section, we summarize the latest advances in emerging therapeutic strategies targeting extracellular histones for the management of these critical or hyperinflammatory diseases. Furthermore, we summarize the mechanisms of these anti-histone strategies in **Figure 2**.

### 3.1 Heparin and its derivatives

#### 3.1.1 Heparin

Heparin, a negatively charged glycosaminoglycan derived from porcine intestine, has been used as an anticoagulant for decades (113). In addition to anticoagulation, heparin also reduces the release of inflammatory molecules, such as interferon (IFN)-γ, TNF-a, IL-6, and IL-8, via the inhibition of NF-κB signaling and the cleavage of complement proteins and ultimately alleviates the inflammatory response *in vivo* (114). As much evidence shown, heparin has a naturally strong affinity for positive toxins such as histones due to its high negative charge density, thus, conditions such as sepsis, COVID-19, intestinal microcirculatory dysfunction and pancreatitis may benefit from heparin administration (114–120).

Numerous data collectively showed that heparin could prevent histone-mediated cytotoxicity, hyperinflammation, and organ injury by forming avirulent heparin-histone complexes (115, 121–124). For instance, unfractionated heparin (UFH) treatment significantly reduced the level of histone-induced inflammatory markers such as IL-6, IL-8, tissue factor and C3a in whole blood (124). Zhu et al. found that the instant intravenous administration of 100 IU/kg/h UFH significantly ameliorated intestinal microcirculation dysfunction in both LPS- and histone-induced endotoxemic rat models by antagonizing histones (115, 119). Jiang et al. indicated that pretreatment with UFH (250 U/kg) significantly inhibited histone-induced alveolar macrophage activation and alleviated lung damage in LPS-induced ARDS murine model (30). Fu et al. reported that UFH (400 U/kg) significantly mitigated histone-induced inflammatory exudation, hyperpermeability, and low expression of syndecan-1 in the lung (109).

In addition to prophylactic treatment or cotreatment with stimuli in *in vitro* and *in vivo* studies, Wang et al. further investigated whether postsurgical heparin interference was still beneficial to histone-induced organ damages (118). In this study, UFH was subcutaneously injected into C57BL/6J mice 4 h after cecal ligation and puncture (118). The UFH-treated group showed a significant decrease in serum histone concentration and kidney injury, as evidenced by reduced expression of neutrophil gelatinase-associated lipocalin, kidney injury molecule-1 and inflammatory factors and less sepsis-induced tissue edema and apoptosis in kidney (118). Similarly, Li et al. found that intravenous injection of 400 U/kg UFH 1 h or 6 h after treatment with 50 mg/kg histone successfully alleviated histone-induced lung injury and pulmonary edema, improved histone-induced endothelial injury and highly procoagulant phenotype, and decreased mortality (120). Additionally, Longstaff et al. reported that the particular combination of heparins and histones might attenuate the anticoagulant effects of heparins (122).

It is well-known that histones could promote fibrinolysis (100, 125, 126). However, the effect of heparin on histone-induced fibrinolysis inhibition remains controversial. Locke et al. reported that therapeutic doses of low-molecule-weight heparin (LMWH) could inhibit histone-fibrin crosslinking and attenuate the delayed effects on histone-induced fibrinolysis (100). However, Komorowicz et al. inversely indicated that the histone-induced inhibition of fibrin lysis by plasmin could not be neutralized by or even exacerbated by polyanions such as heparins and short polyphosphates (125). Administration of LMWH (4000 U/0.4 mL) or aspirin both reduced placenta-mediated pregnancy complications, but only LMWH reversed the histone-induced inhibition of invasion, suggesting that the protective effect of LMWH mainly depended on the direct neutralizing combination of histones and LMWH rather than the anticoagulatory structure (127). Heparin treatment may also reduce the release of extracellular histones. For instance, UFH administration (400 U/kg, 30 min prior to LPS stimulation) was found to diminish the concentration of extracellular histones in a LPS-induced neonatal ARDS mouse model (128).

Although heparin showed extremely strong affinity with histones, which resulted in a significant improvement in histone-induced damage, the specific safety threshold of heparin use in animal models was still unclear. It is widely accepted that the administration of high dose of heparins increases the risk of bleeding, which limits their further clinical application. Sun et al. reported that intraperitoneal injection of 250 U/kg heparin significantly relieved alveolarization and vascular development, reduced NETosis, decreased the levels of inflammatory cytokines such as TNF-α, IL-1β and IL-6, and enhanced the survival rate in both hyperoxia-induced bronchopulmonary dysplasia and histone-induced lung injury murine models without increasing risk of alveolar capillary bleeding (129). However, a higher dose at 500 U/kg showed no effect on reducing NETosis or inhibiting inflammatory reactions but did result in alveolar capillary bleeding (129). In contrast, Iba et al. investigated that both heparin (350 or 700 U/kg) and LMWH (2.0 or 4.0 mg/kg), rather than argatroban, attenuated histone-induced liver and kidney dysfunction and improved the survival rate (116). Additionally, a high concentration of heparin (700U/kg) could further inhibit the interaction between histones and platelets (116). Finally, it is also of great interest whether additional benefits can be gained from combination treatment with low doses of heparins and other anti-histone strategies. Medeiros et al. compared the efficacy of DNase I monotherapy, LMWH monotherapy, and combination therapy (130) and reported that the mono-administration of DNase I and LMWH improved the survival of cecal ligation and puncture-induced septic mice compared with that of saline-treated mice (81.8% vs. 83.3% vs. 38.7%, respectively) (130). Notably, the co-treated group exhibited only a small improvement in survival, suggesting that there may be a negative drug-drug interaction between DNase I and LMWH (130).

#### 3.1.2 Nonanticoagulant heparins and heparinoids

Sharma et al. compared the binding affinity of heparin and its derivatives with different molecular weight and structural characteristics for histone subunits by using biolayer interferometry technology and further investigated their ability to attenuate histone-mediated cytotoxicity, procoagulant activity, and impairments in activated protein C (APC) generation (131). As shown in **Table 1**, all four types of heparin variants showed inversive histone-mediated impairment of APC generation, which indicated that this ability was size- and anticoagulant-independent (131). Reciprocally, the capacity to neutralize histone-induced cytotoxic and procoagulant effects had a strict requirement for a molecular weight above 1.7 kDa (131). Likewise, Wang et al. used 8 μL/g non-anticoagulant heparin intraperitoneally, which reduced liver injury, and fibrogenesis in CCl4-induced liver fibrosis murine models, confirming that the anticoagulant structure was not necessary to neutralize histones (112). Sevuparin, a heparinoid in which the high-affinity antithrombin III-binding pentasaccharide had been removed, significantly reduced group A streptococcal-induced plasma leakage and endothelium activation (81). However, unlike heparin and its derivatives, sevuparin has a compromised ability to inhibit neutrophil adhesion and degranulation (81).

**Table 1:** Heparin variants and their histone-affinity properties.

		UFH	LMWH	Vasoflux (LMWH)	Fondaparinux
Molecular weight		~15 kDa	~5 kDa	~5 kDa	1.7 kDa
Dosage		250 U/kg/q12 h	200 U/kg/24h	200 U/kg/24h	7.5 mg/q24h
Anticoagulant ability		(+)	(+)	(-)	(+)
Histone affinity	H1	2.88×10^4^ ± 9.72×10^3^	4.10 × 10^4^ ± 2.95 × 10^4^	6.67 × 10^4^ ± 3.33 × 10^4^	1.48 × 10^7^ ± 3.98 × 10^6^
	H2A	<1.00 ± 0	<1.00 ± 0	<1.00 ± 0	4.71 × 10^7^ ± 2.02 × 10^7^
	H2B	3.14 × 10^1^ ± 3.04 × 10^1^	<1.00 ± 0	<1.00 ± 0	3.06 × 10^6^ ± 1.97 × 10^6^
	H3	<1.00 ± 0	<1.00 ± 0	<1.00 ± 0	1.53 × 10^7^ ± 6.26 × 10^6^
	H4	<1.00 ± 0	<1.00 ± 0	<1.00 ± 0	8.11 × 10^10^ ± 2.87 × 10^10^
Anti-cytotoxic		Appreciable, Dose-dependent	Appreciable, Dose-dependent	Appreciable, Dose-dependent	Modest, Dose-dependent
Anti-procoagulant		Appreciable, Dose-dependent	Appreciable, Dose-dependent	Appreciable, Dose-dependent	Appreciable, Dose-dependent
Impairment of APC generation		Appreciable, Dose-dependent	Appreciable, Dose-dependent	Appreciable, Dose-dependent	(-)

Antithrombin-affinity chromatography of UFH yields no-anticoagulant or low-anticoagulant heparin molecules. For instance, Reutelingsperger et al. demonstrated that M6229, a low-anticoagulant fraction of UFH obtained by affinity chromatography employing immobilized antithrombin, significantly protected a rat model of acute hyperinflammation from histone-induced liver injury, kidney dysfunction, and mortality (132).

Additionally, the structural requirement of sulfation, which took part in maintaining a negative charged construction, was investigated by Hogwood et al. (124). In this study, heparin and 4 selectively desulfated heparins, 2-O-desulfated, 6-O-desulfated, N-desulfated-re-N-acetylated, and fully desulfated heparin, were added to whole blood from healthy volunteers with or without 50 μg/ml of histones (124). The results showed that both 2-O-desulfated and 6-O-desulfated retained different degrees of anticoagulant activity (90% vs 75%) compared to unmodified heparin, and all selectively desulfated heparins, except fully desulfated heparin, significantly reduced histone-induced responses in both inflammatory reactions and complement activity, suggesting that 1) the position of sulfur modification was significant for the change of heparin anticoagulant activity and 2) negatively charged sulfate groups were important for anti-histone treatment (124). Moreover, N-desulfated-re-N-acetylated heparin, which has a neutral acetyl group, occupied the positive sites exposed after N-desulfation and restored the anti-histone capacity, indicating that there might be other pathways involved in heparin-induced anti-histone treatment in addition to the combination (124).

### 3.2 Chondroitin sulfate and its derivatives

**Figure 3:**
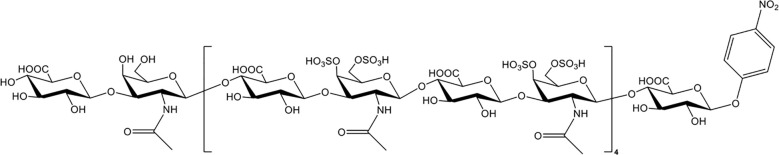
The chemical structure of CS-E 19-mer.

Chondroitin sulfate (CS), which is the side chain of proteoglycans, is widely distributed on the cell surface and in extracellular matrices. Nagano et al. reported that the administration of CS significantly reduced liver and renal injuries, thrombocytopenia, and platelet/leukocyte aggregation in histone-infused rats and exerted protective effects on vascular endothelial cells against histone-induced toxicity *in vitro* (133). Accordingly, Li et al. enzymatically synthesized CS-E nonadecasaccharide (CS-E 19-mer), as shown in **Figure 3** (134). The results showed that the CS-E 19-mer had a tight binding affinity for histones, with a binding affinity constant of 4.47 × 10^-8^ detected by surface plasmon resonance technology (134). CS-E 19-mer treatment alleviated LPS-induced vascular hyperpermeability, improved kidney and liver functions, and reduced mortality from 92% to 30% in LPS-induced septic mice (134). Notably, compared with heparins, CS and the CS-E 19-mer scarcely affected coagulation regulation, suggesting that CS and its variants could be novel agents for lethal systematic disorders at risk for hemorrhage (133).

### 3.3 Natural plasma proteins

Natural plasma proteins, such as albumin, C-reactive protein (CRP), osteopontin, and fibrinogen, can also significantly interact with histones in a charge-dependent manner to prevent the deleterious effects of circulating histones. In this section, we mainly discuss the latest advances in the use of natural plasma proteins as potential histone neutralizers.

#### 3.3.1 Albumin

Early in 1961, it was reported that calf thymus histones can bind to negatively charged albumin through electrostatic interactions (135). Lam et al. first investigated the role of serum albumin in preventing histone-induced platelet activation and aggregation using flow cytometry and aggregometry (7). The results showed that serum albumin significantly inhibited histone-induced platelet activation and aggregation in a dose-dependent manner, which could be remarkably reduced by surface neutralization of albumin through the modification of carboxyl groups in the albumin molecule (7). More recently, Iba and colleagues found that physiological levels of albumin significantly attenuated histone H3-mediated vascular endothelial cell death *in vitro* (136). However, clinical evidence that routine albumin administration may improve the clinical outcomes of critically ill patients with elevated plasma circulating histones is still lacking.

#### 3.3.2 Pentraxin-3

Pentraxin-3 (PTX3), an acute-phase protein that belongs to the long-chain pentameric protein superfamily, is a pattern recognition receptor involved in the regulation of the innate immune response (137). It is widely accepted that plasma PTX3 levels are significantly associated with disease severity in patients with sepsis (138), autoimmune diseases (139), and cardiovascular diseases (140). In 2014, Daigo et al. reported that PTX3 interacted with histones to form histone-PTX3 aggregates, and that PTX3 protected endothelial cells from histone-mediated cytotoxicity both *in vitro* and *in vivo* (141). Additionally, PTX3 administration substantially decreased mortality and massive lung hemorrhage in mice infused with a high dose of histone without affecting platelet function, which is a distinct feature of PTX3 compared with other reported anti-histone molecules, such as anti–histone H4 antibody, heparin, CRP, and recombinant thrombomodulin.

#### 3.3.3 C-reactive protein

C-reactive protein (CRP), a major acute-phase protein, is involved in both innate and adaptive immunity against bacterial infection. Abrams et al. showed that CRP could compete with phospholipid-containing liposomes to form CRP-histone complexes in serum from patients with both elevated CRP and histones (142). *In vitro*, CRP significantly alleviated histone-induced endothelial cell damage, permeability increase, platelet aggregation, and coagulation activation (142). *In vivo*, 10 mg/kg CRP markedly protected mice challenged with lethal doses of histones (75 mg/kg) from lung edema, hemorrhage, thrombosis, and mortality (142). In addition, Hsieh et al. demonstrated that CRP significantly reduced histone-induced neutrophil respiratory burst responses (143). Therefore, CRP-mediated detoxification of circulating histones might be a generic host defense mechanism against excessive inflammation in humans. However, whether an elevated CRP level will bring a net anti-inflammatory benefit in patients with hyperinflmmatory conditions such as interstitial lung disease, cytokine release syndrome following post CAR-T cell therapy, sepsis, and severe COVID-19 remains unknown.

#### 3.3.4 Osteopontin

Osteopontin (OPN), a highly anionic molecule secreted by epithelial cells, can also modulate the proinflammatory and cytotoxic properties of circulating histones. Kasetty et al. found that OPN bound to histones with high affinity *in vitro* (Kd=2.8×10^-7^ for histone H3.1 and Kd=1.4×10^-8^ for histone H4 determined by surface plasmon resonance) and that the histone–OPN complex levels in the bronchoalveolar lavage fluid of ARDS patients were significantly higher than those in the bronchoalveolar lavage fluid of healthy individuals (144). Accordingly, OPN treatment inhibited the histone-induced cytotoxic and hemolytic effects of histones, significantly reducing histone-induced epithelial cell cytotoxicity, endothelial cell hyperpermeability, and hemolysis *in vitro* and protecting mice from histone-induced acute liver injury *in vivo*, as evidenced by a significant increase in the incidence of necrosis of hepatocytes in OPN^-/-^ hepatic injury mice induced by ischemia–reperfusion (145).

#### 3.3.5 Cl esterase inhibitors

Cl esterase inhibitor (ClINH), an endogenous acute-phase protein, is the main physiologic inhibitor of the contact phase and the kallikrein-kinin system (146). In 2012, Igonin et al. conducted a randomized controlled study involving 62 sepsis patients to assess the impact of high-dose ClINH administration (12,000 U) on the systemic inflammatory response and survival (147). Their results showed that ClINH infusion in sepsis patients was significantly associated with reduced all-cause mortality (12% vs. 45% in controls) as well as sepsis-related mortality (8% vs. 45% in controls) over 28 days (147). Since then, increasing evidence has shown that ClINH can also neutralize histone-induced toxicity via tight combination with histones due to its glycosylation-dependent overall negative charge (148). C1INH was found to bind all histone types, independent of its protease inhibitory activity (148). Moreover, C1INH treatment inhibited the cytotoxic activity of extracellular histones *in vitro* and protected mice against histone-evoked lung injury and death. As histone-mediated hyperinflammation and organ injury may occur in numerous critical illnesses, the application of C1INH may provide a new therapeutic option in disease states characterized by excessive inflammation and cell death.

#### 3.3.6 Clusterin

Clusterin (CLU) is a ubiquitous extracellular protein that chaperones misfolded proteins and promotes their removal. Recently, CLU was found to be dramatically decreased in patients with sepsis (95.0 ± 67.5 in non-survivals vs. 133.1 ± 69.5 µg/mL in survivals) (149). Augusto et al. reported that CLU could bind to histones with a dissociation constant of 4-6 nM in a dose-dependent manner (149). Pretreatment with CLU inhibited the inflammatory, thrombotic, and cytotoxic properties of circulating histones *in vitro* (149). Furthermore, intravenous CLU supplementation significantly improved survival in lethal dose of histone (100 mg/kg)-induced murine models, murine endotoxemia models and CLP-induced septic models (149). The identification of CLU as an endogenous regulator of histone activity provides another example of the host’s intrinsic ability to counter or quench ongoing proinflammatory and antimicrobial defense mechanisms to maintain homoeostatic control.

#### 3.3.7 Fibrinogen

Fibrinogen, an acute phase protein, could functionally sequester histones as complexes to eliminate the cytotoxic effects of histones and cluster on the surface of ionomycin-stimulated neutrophils to delay NETosis in a β2-integrin-dependent manner, thus significantly maintaining the viability of neutrophils (150). More recently, Toh et al. (151) demonstrated that fibrinogen bound histones through its D-domain with high affinity (calf thymus histones KD=18.0 ± 5.6 nM, histone 3 KD=2.7 nM ± 0.8 nM, and histone 4 KD=2.0 ± 0.7 nM) and thus significantly reduced histone-induced endothelial damage and platelet aggregation. In a cohort of critically ill patients, the authors further found that a histone-to-fibrinogen ratio≥6 on admission was associated with moderate-severe thrombocytopenia and independently predicted mortality. Fibrinogen buffers the cytotoxic properties of circulating histones.

#### 3.3.8 Inter-α inhibitor protein

Inter-α inhibitor protein (IAIP), an endogenous plasma serine protease inhibitor, carries a CS moiety in the light chain where the light chain covalently links to 2 heavy chains (152). Chaaban et al. revealed that IAIP, particularly high-molecular-weight hyaluronan and negatively charged CS which showed natural affinities for histones, could significantly reduce histone-induced cytotoxicity via reducing histone-induced calcium influx, platelet aggregation, prothrombinase activity in a dose-dependent manner *in vitro* (152). Pre-injection of IAIP counteracted histone-induced thrombocytopenia, prolonged bleeding time, systemic inflammation and organ injury, as evidenced by a significant abrogation of P-selectin expression, fibrin deposition and neutrophil accumulation in the lung and decrease plasma levels of proinflammatory cytokines (IL-1β, IL-6, TNF-α), chemokines, and anti-inflammatory IL-10 (152).

### 3.4 Other histone neutralizers

#### 3.4.1 Methyl β-cellobioside per-O-sulfate

**Figure 4:**
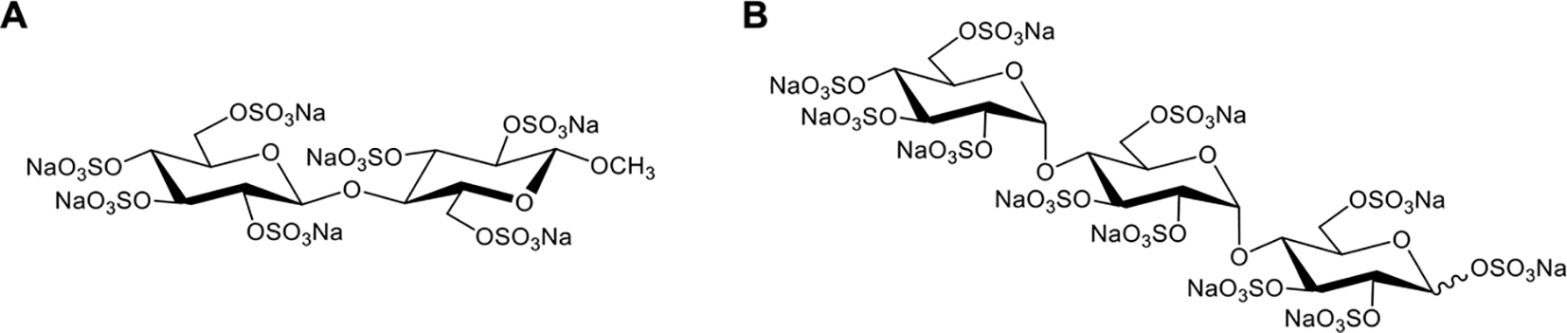
Chemical structures of mCBS **(A)** and MTS **(B)**.

In 2020, Meara et al. reported that a highly sulfated hexose disaccharide was the minimum structure for small polyanions (SPAs, ~0.9–1.4 kDa) to inhibit histone-mediated cytotoxicity as effectively as heparins (85). In this study, three SPAs, namely, cellobiose per-O-sulfate (CBS), methyl β-cellobioside per-O-sulfate (mCBS), and maltotriose per-O-sulfate (MTS), were found to inhibit the cytotoxic, platelet-activating and erythrocyte-damaging effects of histones by electrostatically interacting with histones. SPAs could also improve the stability of lipid bilayers and reduce histone-induced intracellular calcium overload *in vitro* (85). Furthermore, both mCBS and MTS (the chemical structures are shown in **Figure 4**) significantly improved liver, cardiac, and kidney injury with less cell death in the tissue in histone-infused, CLP and cardiac ischemia-reperfusion injury murine models. However, the authors found that MTS, the most potent neutralizer of DNA-free histones in this study, was not effective in protecting mice from NET-mediated cardiac ischemia-reperfusion injury and peritonitis-induced sepsis. Therefore, mCBS ahead of MTS was chosen as the SPA for clinical development. Importantly, mCBS, which was well tolerated in rats and dogs when continuously infused at 125 mg/kg/h for 14 days, had excellent safety and minimal anticoagulant activity; that is, mCBS was 110-fold less effective than LMWH and 764-fold lower than UFH. More recently, Shah et al. reported that mCBS significantly reduced histone-mediated cardiomyocyte death and infarct size in isolated murine myocardial ischemia–reperfusion models (16). Similarly, Ge et al. indicated that intravenous injection of 100 mg/kg mCBS mitigated LPS-induced lymphocyte recruitment, neutrophil infiltration, inflammatory cytokine release, and pulmonary edema and significantly improved oxygenation function in LPS-induced ARDS rat models (153). In 2023, a randomized placebo-controlled experimental study further assessed the effects of mCBS on the severity and outcome of a peritonitis-induced sheep sepsis model. Twenty-four mechanically-ventilated female sheep were randomized into three groups: control, early treatment, and late treatment (n = 8 each). mCBS was given as a bolus (1 mg/kg) followed by a continuous infusion (1 mg/kg/h) just after sepsis induction in the early treatment group, and 4 h later in the late treatment group. The results showed that mCBS-treated animals required significantly less norepinephrine to maintain a mean arterial pressure between 65 and 75 mmHg than did controls and had lower creatinine, lactate, and IL-6 levels, which were associated with reduced changes in plasma H3.1 nucleosome levels (p = 0.02). Together, this encouraging preclinical evidence indicates that neutralization of extracellular histones with mCBS may represent a new therapeutic approach for multiple histone-mediated critical illnesses, including sepsis, severe COVID-19, and ARDS.

#### 3.4.2 Magnesium

Magnesium ion, the second most abundant intracellular cation after potassium, may possess anti-inflammatory and antioxidant properties (154). Hypomagnesemia is commonly observed in 22.2% of septic patients and can lead to multiple life-threatening complications, such as malignant arrhythmia, coronary artery spasm, and cardiac arrest. In 2023, Gu et al. conducted a retrospective cohort study using the Medical Information Mart in Intensive Care-IV database to investigate the association of magnesium administration with mortality and organ support in critically ill septic patients (155). The results showed that magnesium sulfate use was significantly associated with lower in-hospital mortality and a lower need for renal replacement therapy. Most recently, Zhong et al. studied the role of magnesium supplementation in preventing histone-mediated macrophage damage, coagulation dysfunction, and lung injury in both septic patients and animal models (156, 157). Their clinical data showed that the level of circulating histones correlated negatively with both monocyte and platelet counts in septic patients and that low magnesium levels were associated with low monocyte levels in such patients. In histone-induced mice models, magnesium administration significantly improved survival and attenuated histone-mediated endothelial cell injury, coagulation dysfunction, and lung histopathological damage. Magnesium can further protect macrophages from apoptosis and defective bacterial phagocytosis through the PLC/IP3R/STIM-mediated calcium signaling pathway and therefore significantly reduce the bacterial load in a CLP+ histone-induced mouse model. Accordingly, well-designed prospective randomized controlled studies are needed to further verify the protective effect of magnesium supplementation in septic patients with high plasma levels of circulating histones.

#### 3.4.3 Suramin

**Figure 5:**
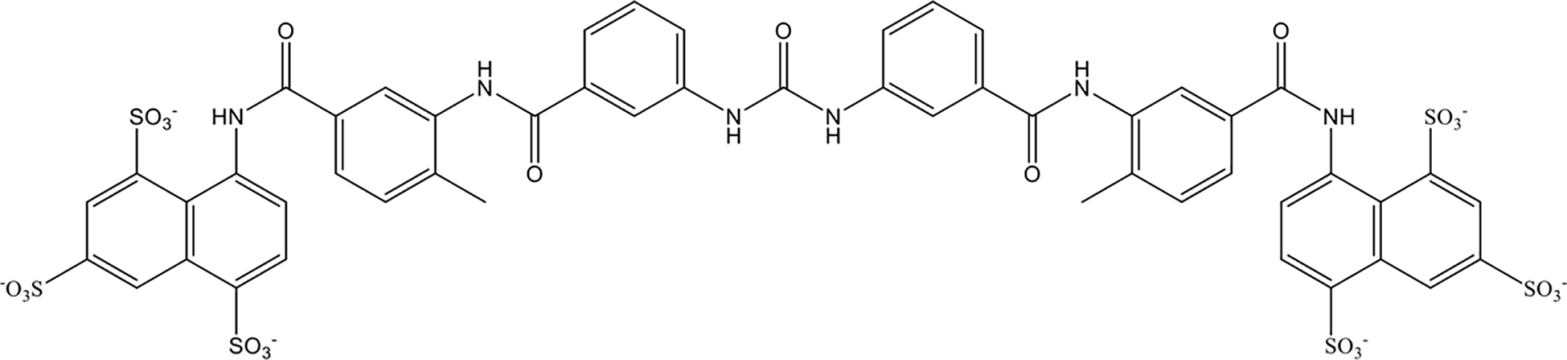
Chemical structure of suramin.

Suramin is a multifunctional polyanionic drug that has been used for a century to treat the first stage of acute human sleeping sickness caused by *Trypanosoma brucei rhodesiense*. As shown in **Figure 5**, suramin is highly negatively charged owing to the presence of sulfate groups in its molecular structure. In 2023, Villalba et al. demonstrated that suramin avidly bound to extracellular histones with a dissociation constant of 250 nM through stable electrostatic interactions between the sulfate groups on suramin and hydrogen bonds in the histone octamer. *In vitro*, suramin blocked histone-evoked thrombin generation in endothelial cell cultures and vasodilatory dysfunction in isolated mouse vessels (158). Suramin administration (50 mg/kg) significantly protected mice from lethal doses of histones (75 mg/kg) by decreasing lung endothelial cytotoxicity, lung edema, and intra-alveolar hemorrhage *in vivo* (158). However, suramin has less affinity for citrullinated histones from neutrophil extracellular traps. Together, these data demonstrate the therapeutic potential of suramin in critically ill patients characterized by elevated circulating histone levels.

#### 3.4.4 Histone inhibitory peptide

In 2019, Silvestre-Roig et al. first revealed that extracellular histone H4 mediated membrane lysis of smooth muscle cells and thus contributed to arterial tissue damage and inflammation. Accordingly, they synthesized a histone inhibitory peptide that could form stable complexes with the H4N-terminus, which was found to attenuate histone-mediated smooth muscle cell death and stabilize atherosclerotic lesions (26). They further investigated that the continuous administration of histone inhibitory peptide significantly increased the stability of plaques but did not alter neutrophil recruitment in spontaneous atheroprogression murine models (26). More recently, the authors further developed another novel peptide inhibitor, namely, cyclical histone H2A interference peptide, which bound to NET-resident histone H2A to block monocyte adhesion and consequently reduced atheroprogression during endotoxemia in hypercholesterolemic mice (159). These new synthetic peptides may provide a new direction for the development of novel anti-histone drugs and the management of cardiovascular diseases and other histone-related chronic inflammatory diseases.

#### 3.4.5 RNA aptamers

Aptamers, a group of chemically stabilized nucleic acid drugs, are synthetic RNA or DNA oligonucleotide ligands that have high affinity and specificity for their targets (160). Unlike proteins, aptamers are redox-, pH- and temperature-insensitive, small-sized, and economical to produce (161). In this regard, Urak et al. developed an RNA aptamer-based anti-histone therapeutic strategy to selectively neutralize extracellular histones implicated in MODS. Nuclease-resistant, 2′ fluoro-modified RNA aptamers that had a high affinity for histones were first developed by exponential enrichment. By using surface plasmon resonance, the dissociation constants between the tailored RNA aptamers (KU7 and KU9) and histone H4 were determined to be 4.01 and 1.51 nM, respectively, while no significant binding between the RNA aptamers and human albumin was observed, confirming the specificity of the tailored RNA aptamers for histones vs. serum proteins. On the one hand, the obtained RNA aptamers significantly inhibited histone-mediated platelet aggregation and endothelial cell death *in vitro*. On the other hand, RNA aptamers not only alleviate calf thymus histone-induced TLR activation, as measured by IL-6 production but also reduce edema, thrombi, hemorrhage, and inflammatory factor IL-6 expression in the liver, lung and spleen in histone-infused murine models (160). Similarly, Lei et al. showed that a tailored RNA aptamer (KU7) could prevent histone-induced proinflammatory cytokine production, endothelial dysfunction and platelet activation *in vitro* (162). In a histone-mediated acute lung injury mice model, inhalation of KU7 prevented increased pulmonary vascular permeability and alleviated histone-evoked alveolar damage, as evidenced by reduced neutrophil infiltration, alveolar destruction, and interstitial edema in KU7-treated lungs (162). Together, these data highlight the potential safety of RNA aptamers for targeting extracellular histones while minimizing unwanted off-target effects.

#### 3.4.6 Defibrotide

**Figure 6:**
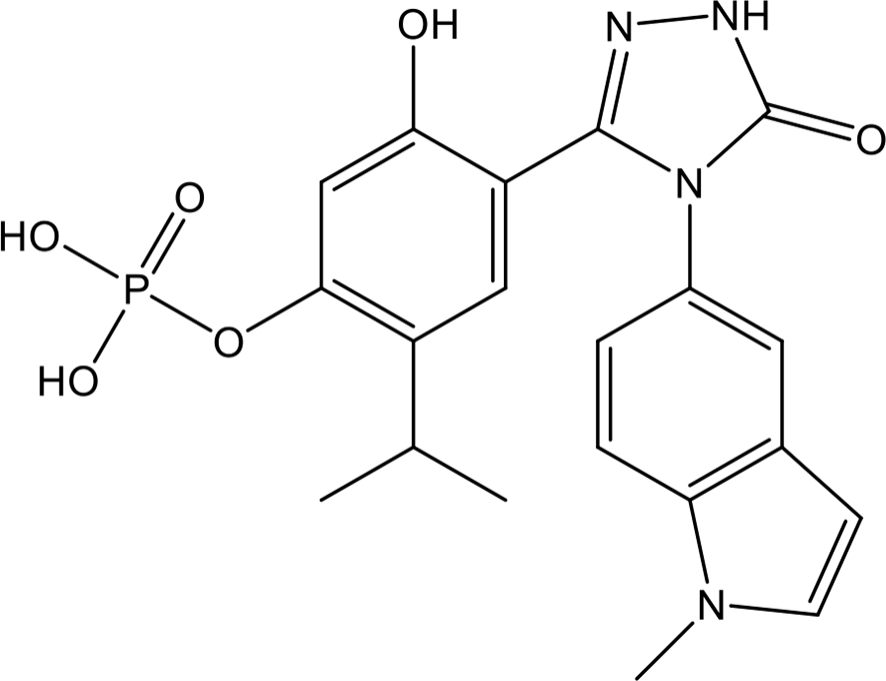
Chemical structure of defibrotide.

As shown in **Figure 6**, defibrotide is a polydisperse mixture of predominantly single-stranded polydeoxyribonucleotide sodium salts that is produced via controlled depolymerization of porcine intestinal DNA (163). It is well established that defibrotide has antithrombotic, fibrinolytic, anti-inflammatory, antioxidative, and antiadhesive effects. Unlike heparin and other common anticoagulants, defibrotide has no systemic anticoagulant effects, making it safer to use in critically ill patients with intrinsic coagulation disorders. Recently, Shi et al. reported that defibrotide directly and tightly bound to histone H4 through charge-charge interactions with an equilibrium dissociation constant of 53.5 nM, as detected by surface plasmon resonance (164). Accordingly, defibrotide abolished histone-induced endothelial activation, endothelial cell death, and hyperpermeability of the endothelial layer *in vitro* and countered histone-mediated endothelial activation and venous thrombosis in large-vein thrombosis murine models (164). These data provide insights into the potential role of defibrotide in protecting the endothelium from thrombo-inflammation with potential therapeutic potential for histone-mediated critical illnesses.

#### 3.4.7 Polyphenol-modified proteins

Protein modification also helps to neutralize histones. Recently, Itakura et al. figureported that proteins incubated with oxidized vitamin C can function as histone-binding ligands, inhibit the binding of plasminogen to histone H2B and regulate the recruitment of monocytes/macrophages to sites of inflammation. In light of this finding, Yamaguchi et al. further screened 7 polyphenol-modified proteins as a source of histone ligands. The authors demonstrated that the obtained polyphenol-modified proteins had strong affinities for the N-terminal tail region of histones to form aggregates and thus reduced histone-mediated endothelial cytotoxicity (165). In the future, more studies are needed to evaluate the formation of protein-bound polyphenols *in vivo* and investigate the contribution of this unique molecule to histone-related diseases.

#### 3.4.8 Thrombomodulin

Thrombomodulin, expressed on the surface of the endothelium, is a cofactor that regulates intravascular coagulation (166, 167) and consists of 5 domains, while the N-terminal lectin domain is capable of exerting anti- inflammatory effects (166) and combining with some DAMPs (168). Akatsuka et al. reported that both recombinant thrombomodulin and the N-terminal lectin domain could significantly reduce H3 release, neutralize extracellular histones to decrease H3 levels and improve the prognosis of CLP-induced septic murine models (169). Interestingly, no significant difference was found between the recombinant thrombomodulin-treated group and the N-terminal lectin domain-treated group, indicating that the N-terminal lectin domain was the core domain of recombinant thrombomodulin for anti-histone function (169).

#### 3.4.9 Nanomedicines

**Figure 7:**
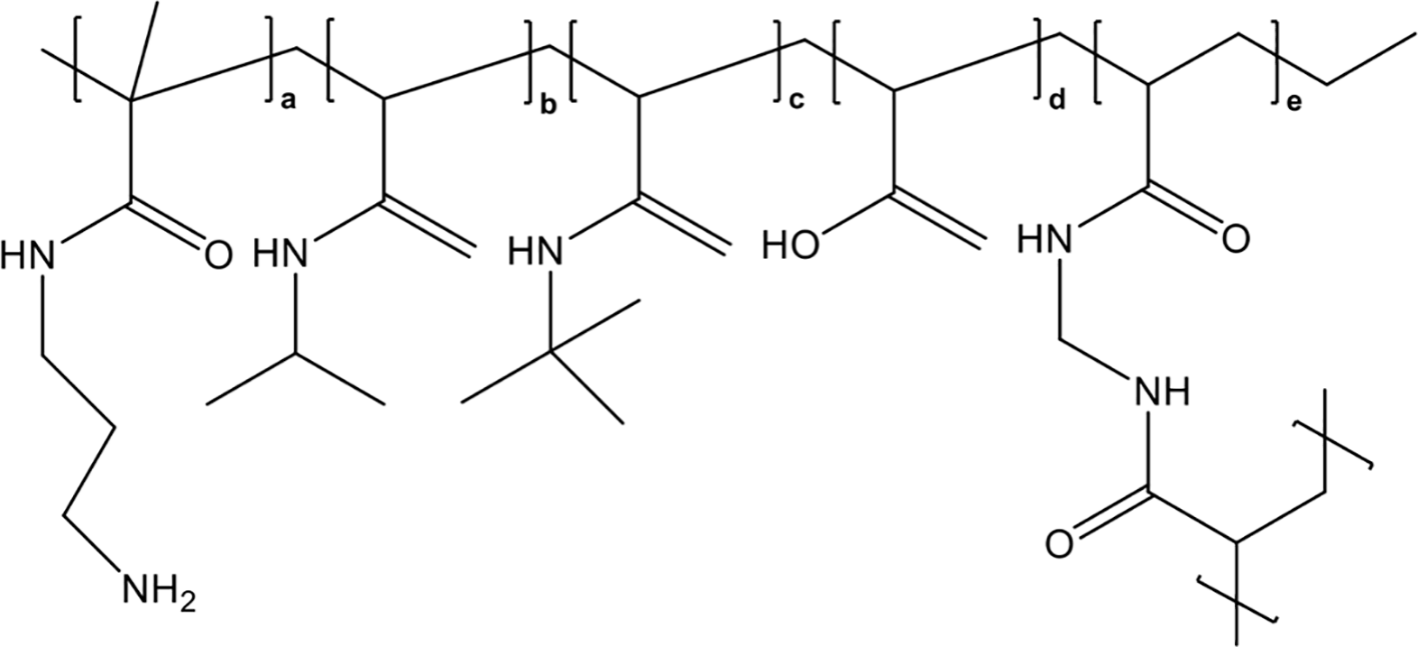
Chemical structure of HNPs.

Synthetic, nonbiological polymer nanomedicines to capture and neutralize extracellular histones are also promising therapeutic agents for sepsis therapy. Koide et al. first attempted to conjugate histone-capturing synthetic linear copolymers to a lipid nanoparticle (a highly biocompatible drug delivery agent) to achieve specific histone neutralization in the bloodstream circulation (170). However, these lipid nanoparticles had a low histone capture capacity and tended to aggregate after histone capture. To overcome these limitations, the same research group developed polyethylene glycol (PEG)-modified hydrogel nanoparticles (PEGHNPs), which showed greater affinity for histones and longer remaining time in the bloodstream compared with naked hydrogel nanoparticles (HNPs), whose chemical structure was shown in **Figure 7**, and thus significantly improved the histone adsorption efficiency of HNPs (171). They further explored the curative effects of optimized PEGHNP12, which had the longest circulation time (150 times longer than that of naked HNPs) and high affinity for histones. The results indicated that PEGHNP12 could selectively neutralize both circulating and cell-surface-captured histones without strong affinity for common, abundant proteins in plasma, such as albumin and fibrinogen. Moreover, PEG decoration inhibited histone-induced interactions with cell surfaces and cellular uptake compared with those of naked HNPs, and PEGHNP12-histone complexes exhibited less aggregation after the formation of PEGHNP12-histone complexes. Most importantly, pretreatment with 10 mg/kg PEGHNP12 before histone stimulation (75 mg/kg) significantly inhibited platelet aggregation and migration into the lungs, and improved the survival rate 2-fold compared with those in the naked HNPs group and saline group. Similarly, compared with saline and naked HNPs, PEGHNP12 significantly decreased mortality in LPS-induced septic murine models compared with saline and naked HNPs groups during a 20-hour observation period.

### 3.5 Monoclonal anti-histone antibodies

After being released into the circulation, histones cause tissue damage and trigger a vicious cycle via direct contact or incurring internal circulation disorders. Hence, efforts had been put in investigating the various monoclonal anti-histone antibodies recently (10, 40, 68, 172, 173). Deng et al. investigated an anti-histone H3 (citrullinated R2+R8+R17+R26) monoclonal antibody that could completely block the catalyzation by both PAD2 and PAD4 (173). This novel antibody decreased the serum levels of IL-1β and dsDNA and protected against LPS-induced acute lung injury, as assessed by histopathology changes and acute lung injury scores in lethal LPS-induced septic murine models (173). Importantly, compared with the CitH3 mAb (3 Cit), the CitH3 mAb (4 Cit) exhibited better histone binding ability, histopathological changes and survival rate (173). Similarly, Tian et al. indicated that intravenous injection of anin-house-developed CitH3 antibody with four citrulline residues significantly inhibited Capsase-1 activation, attenuated lung injury and improved the survival rate in murine models of CLP-induced septic murine models (68). Abrams et al. produced an anti-histone scFv (ahscFv) that could recognize histones H1, H3 and H4 and significantly decreased histone-mediated cytotoxicity (10). Moreover, pretreatment with 10 mg/kg ahscFv significantly decreased the serum levels of soluble thrombomodulin, thrombin antithrombin, and IL-6 and improved pathological changes and the survival rate in both trauma- and histone-infused murine models (10). Although many studies have reported that anti-histone antibodies can protect against histone-induced cytotoxicity, their efficacy has been found to be inconsistent to some extent. Hence, there is a need for the development of more reliable anti-histone antibodies, and more efforts are needed to provide solid data from *in vivo* animal experiments.

### 3.6 Inhibition of the release of extracellular histones

#### 3.6.1 PAD4 inhibition

Modifications of histones could change the way they interact with cells by affecting their size, charge, and structure (88). For instance, citrullination, the most common but not the only form of histone modification (45), was reported to increase the cytotoxic effect of histones on HUVECs, including significantly increased permeability and triggered NETosis (173). However, the differences in cytotoxicity between native histones and citrullinated histones remain controversial (174). Histone citrullination, which is mainly modified by PAD4, is commonly recognized as the initiator of NETosis and secondary histone release in sepsis (173, 175), ARDS (10), COVID-19 (176), lupus nephritis (177), rheumatoid arthritis (178). Many studies demonstrated that pharmacological or genetic approaches inhibiting PAD4 activity in animal models could help to achieve obvious benefits by reducing histone release and NETosis (43, 179–194).

#### 3.6.2 Short-chain fatty acids

It has been reported that probiotics that colonize the intestine under physiological conditions can produce short-chain fatty acids like acetate, butyrate and propionate, which have anti-inflammatory abilities to some extent (195, 196). An increasing number of experiments showed that the administration of short-chain fatty acids improved the outcomes of acute kidney injury (110, 111, 197). A recent study by Li et al. demonstrated that short-chain fatty acids might improve the *Staphylococcus aureus*-induced inflammatory response and endothelial injury by inhibiting the autophagy of neutrophils, which resulted in decreased NET formation and histone release (198). However, the underlying mechanism remains unclear (198).

#### 3.6.3 Dexmedetomide

Furthermore, Sun et al. reported that dexmedetomide, an α2-adrenoceptor agonist, could alleviate LPS-induced NLRP3 inflammasome recruitment, caspase-1 activation, and astrocyte pyroptosis *in vitro* (67). Dexmedetomide stabilized the cellular integral structure, which resulted in decreased quantification of histones in the culture medium compared with that in the LPS-treated group and improved survival in LPS-induced septic rats (67).

### 3.7 Promotion of the degradation of extracellular histones

It has been proposed that the noncleaved histone contributes to tissue damage (34). Proteolysis of histones seems to be an advisable treatment. To date, several histone scavengers have been reported to cleave histones into fragments to abolish their damaging abilities. In this section, we mainly discuss the use of histone-degrading agents for the treatment of histone-related diseases.

#### 3.7.1 Factor VII-activating protease

Factor VII-activating protease (FSAP), a serum serine protease, is widely involved in regulating hemostasis, inflammation, fibrinolysis, and tissue remodeling. Marsman et al. indicated that after the incubation of serum with histones, endogenous FSAP was activated, suggesting that histones were also involved in FSAP activation and promoted fibrinolysis (126, 199, 200). Additionally, FSAP could cleave the N-terminal tail of free histone H3 to reduce histone-mediated cytotoxicity *in vitro* and decreased free serum histone levels in both septic baboons and patients with meningococcal sepsis (200). Similarly, Cui et al. found that the serine protease domain of FSAP could significantly attenuated histone-induced hyperpermeability, reduced the redistribution of junctional proteins, and abolished histone-induced upregulation of TLR-2 expression *in vitro* (61). Notably, FSAP caused multisite cleavage of histones, not just N-terminal cleavage, suggesting thorough degradation of histones by FSAP (61). However, solid data from *in vivo* animal experiments are needed to support the use of exogenous FSAP to cleave histones for sepsis treatment.

#### 3.7.2 Activated protein C

Protein C, a vitamin K-dependent protein and a member of the protein C system that is synthesized by hepatocytes, is converted to APC by the thrombomodulin-thrombin complex on the endothelium (9). Xu et al. reported that coinjection of APC significantly relieved histological injuries in the lung, ameliorated renal function, and improved the survival rate in both histone-infused murine models and ***E. coli***-injected baboons (9). Interestingly, this anti-histone capacity was reported to be mediated by the direct cleavage of histones after binding histones with its densely anionic N-terminal Gla domain rather than by influencing cellular pathways such as PAR1 signaling (9, 94). In addition, Xu et al. indicated that liposomes containing phosphatidylethanolamine enhanced histone cleavage via APC, indicating that phospholipid exposure after injury acted as a potent agonist of the anti-histone effects of APC (9). Healy et al. reported that APC could bind human leukocytes, prevent activated platelet supernatant or phorbol 12-myristate 13-acetate from inducing NETosis, and inhibit histone citrullination in an neutrophil receptors endothelial protein C receptor-, protease activated receptor 3-, and macrophage-1 antigen dependent manner (201). Saffarzadeh et al. further indicated that APC significantly reduced histone-mediated cytotoxicity rather than NET-mediated cytotoxicity, possibly because the formation of complexes between histones and other NET components limited the degradation ability of APC (202). Notably, the routine use of APC is not optimal for the treatment of histone-mediated critical illnesses due to the bleeding side effects associated with the anticoagulant properties of APC. To address this issue, Huckriede et al. used molecular docking and molecular dynamics simulation methods to identify key interacting residues that mediated the interaction between APC and histone H3 and to generate novel optimized APC variants (203). The results demonstrated that the designed APC variants 3D2D-APC and 3D2D2A-APC showed significantly decreased anticoagulant activity, increased binding to histone H3, and a similar ability to proteolyze histone H3 compared with wild type APC. Therefore, it is possible to rationally design APC variants that do not increase the risk of bleeding to treat histone-mediated diseases via the proteolytic reduction of histones.

#### 3.7.3 Cytotoxic T lymphocyte protease granzyme A

Granzyme A (GzmA), also known as a cytotoxic T lymphocyte protease protein, is expressed in the nucleus and is related to caspase-independent cell death when it is introduced into target cells by perforin (204). GzmA targets nuclear proteins like histones and lamins for degradation. However, the anti-histone effect of GzmA on cell death and differentiation has been reported (204, 205). Evidence for the use of GzmA or its variants for anti-histone therapy is sparse.

### 3.8 Targeting histone-related pattern-recognition receptors

As outlined above, cellular pathways activated by histones mainly include TLRs and inflammasome signaling pathways (68). To date, numerous studies indicated that TLR-2/4 inhibitors/antibodies could attenuate histone-associated tissue damage (82, 206), reduce platelet-actin-associated molecules such as P-selectin, PS and FV/Va (94), and relieve inflammation (206). However, these inhibitors could not tackle all types of histone-mediated impairments, such as myocardial ischemia-reperfusion injury (16) and atherosclerosis (26). Thus, exploring the specific mechanism of histone-induced endothelial dysfunction, which may be the initiating factor of tissue damage, would be valuable.

### 3.9 Extracorporeal blood purification for histone removal

Extracorporeal blood purification has been widely used to replace dysfunctional organs and remove exogenous or endogenous toxins in clinical practice for more than six decades. Recently, growing preclinical and clinical evidence has supported that extracorporeal blood purification with adsorptive hemofilters, such as the CytoSorb^®^ hemoperfusion cartridge and oXiris membrane, may exert an immunomodulatory effect by eliminating inflammatory cytokines, endotoxin, and histones in patients with critical illnesses (207, 208). In this section, we will discuss the latest advances in the removal of circulating histones from blood by different hemofilters.

CytoSorb^®^ (CytoSorbents Inc., NJ, USA) is a cylindrical cartridge that is filled with tiny, highly porous, hemocompatible polyvinylpyrrolidone-coated polystyrene-divinyl-benzene copolymer beads with a total surface area of > 40,000 m^2^. It is evident that hemoadsorption with CytoSorb^®^ significantly adsorbs hydrophobic cytokine molecules within the 5–55 kDa molecular weight range (209). Recently, Weber et al. found that a 6-h hemadsorption procedure with a CytoSorb adsorber significantly decreased circulating histone levels in the blood of 22 humans with multiple injuries. *In vitro*, approximately 92% to 99% of histones could be adsorbed by a mini CytoSorb^®^ hemadsorption column, although the specific mechanism of histone adsorption by CytoSorb^®^ is still unknown (210). Future clinical studies need to evaluate the efficacy and safety of CytoSorb^®^ for histone adsorption in patients with multiple types of trauma and other critical illnesses.

Extracorporeal hemoadsorption with heparin-functionalized adsorbents may significantly eliminate circulating HMGB1 and histones to exert their immunomodulatory effects. For instance, Marie et al. found that both Seraph-100 and heparin-immobilized Sepharose beads could result in efficient depletion of histones in septic plasma samples (211).

## 4 Future perspectives

In summary, extracellular histones are crucial contributors for hyperinflammation, platelet aggregation, coagulation disorders, endothelial dysfunction, and organ dysfunction, and level of histones in plasma are correlated with disease severity. Preclinical studies have consistently demonstrated that various methods for reducing free histone levels, especially heparins, anti-histone antibodies and novel negatively charged particles such as mCBS, Defibrotide, Suramin and HNPs, can effectively alleviate inflammation, reduce tissue exudation, relieve multiorgan damage and thus improve prognosis of histone-infused animal models.

It should be noted that there are also several research challenges hampering the use of these histone-targeting therapeutic strategies in clinical practice. First, the widely used methods used to detect histones in serum are compromised in distinguishing free histones, DNA-histone complexes and nucleosomes. Due to the large differences in histone concentration between different models, these encouraging findings should be further investigated under precise detection of histone concentration, which remains a major problem for histone-related studies. To solve this problem, Park et al. developed a miniaturized biosensor that could detect the concentration of citH3 with high speed, high sensitivity, high accuracy, and a large detection range (212). Thålin et al. utilized highly specific monoclonal antibodies and semisynthetic nucleosomes containing citrulline in place of arginine at histone H3 to improve a novel approach that enabled reliable quantification of H3Cit in human plasma, expanding the practicality of citH3 as a disease biomarker (213). Everitt et al. constructed an instantaneous diagnostic system with a 112 ng/mL detection limit that utilized the inherent interactions between histones and DNA (214). After being added to blood samples that were pretreated with dextran to induce red blood cell aggregation, the 147 bp double-stranded DNA wrapped around histones was removed. Additionally, after an incubation period, EvaGreen intercalated into DNA that was not bound to a histone and then fluoresced (214). Thus, an inverse relationship between the fluorescence signal and free histone concentration was observed, enabling the quantification of histones in the sample (214). More recently, Matta et al. developed a multiplex ELISA that combined the use of three antibodies against myeloperoxidase (MPO), citrullinated histone H3 (CitH3), and DNA to detect NETs in serum/plasma with increased specificity (215). Additionally, they designed a novel smear immunofluorescence assay to detect NETs in as little as 1 μL of serum/plasma along with other bodily fluids, which could also visually detect intact structures of NETs with minimal time, reagents, specialized equipment, and/or cost (215). However, immunoassays usually show poor reproducibility, wide error ranges and low concordance between assays due to the variety of antigen-antibody interferences. García-Giménez et al. proposed an absolute quantification method for extracellular histones based on multiple reaction monitoring targeted mass spectrometry (MRM-MS), which used standard curves with different concentrations of light peptides and stable isotopically labelled spike-in peptides to detect circulating H3 and H2A in plasma (14). In this work, the peptides LLLPGELAK and STELLIR were chosen as the best candidates for H2B and H3, respectively, for subsequent spiked-in preparations and posterior MRM-MS measurements in blood samples (14). Using receiver operator characteristic curve analysis, this method showed optimal sensitivities and specificities in detecting H3 (sensitivity = 94.1%, specificity = 90.0%, cut-off value = 574.25 ng/mL) and H2B (sensitivity = 82.4%, specificity = 70.0%, cut-off value = 739.53 ng/mL) (14). They further validated the detection of H2B in 89 patients with sepsis and found that H2B was an optimal biomarker for early-stage diagnosis of septic process (sensitivity = 77.0%, specificity = 89.0%, cut-off value = 0.53 ng/mL) and sepsis classification (sensitivity = 63.2%, specificity = 72.7%, cut-off value = 74.66 ng/mL) (12).

Second, knowledge gaps also appear when only citrullinated histones and nucleosomes are detected and treated in some studies. It is noteworthy that citrullination is not a universal characteristic of histones, and not all histone variants exhibit same cytotoxicity. Many evidence revealed that purified nucleosomes are not toxic to cultured endothelial cells *in vitro*, and injection of 1 mg of megadose in mice did not induce any cytotoxicity or mortality *in vivo*, while injection of 1.25 mg of purifies histones in mice is lethal within 1 h (216). Interestingly, nucleosomes can also induce neutrophil activation with CD66b and CD11b upregulation, and secreted IL-8 in a TLR 2/4/9- and MyD88- independent manner (216). Moreover, nucleosomes have been reported to induce lymphocyte necrosis, which is not found in circulating histones, as evidenced by a significant decrease in lymphocytes in the spleen, while there were no signs that lymphocytes had migrated to other organs after the injection of purified nucleosomes in mice (216). Thus, more explorations are encouraged to accurately determine the concentrations of all variants of free histones.

Third, although studies on anti-histone strategies have increased over the past few decades, comparisons of therapeutic effects between various anti-histone drugs are still lacking. Thus, more efforts are needed to compare the safety and efficacy of currently available histone-targeting therapeutic strategies using *in vitro* studies and *in vivo* animal models, which are valuable for determining the tailored histone-targeting strategy for better management of patients with histone-related critical or inflammatory diseases.
